# Floral homeotic C function genes repress specific B function genes in the carpel whorl of the basal eudicot California poppy (*Eschscholzia californica*)

**DOI:** 10.1186/2041-9139-1-13

**Published:** 2010-12-01

**Authors:** Aravinda L Yellina, Svetlana Orashakova, Sabrina Lange, Robert Erdmann, Jim Leebens-Mack, Annette Becker

**Affiliations:** 1 University of Bremen, Fachbereich 02 Biology/Chemistry, Evolutionary Developmental Genetics Group Leobener Str., UFT, 28359 Bremen, Germany; 2 Department of Plant Biology, University of Georgia, Athens, GA 30602-7271, USA

## Abstract

**Background:**

The floral homeotic C function gene *AGAMOUS *(*AG*) confers stamen and carpel identity and is involved in the regulation of floral meristem termination in *Arabidopsis*. *Arabidopsis ag *mutants show complete homeotic conversions of stamens into petals and carpels into sepals as well as indeterminacy of the floral meristem. Gene function analysis in model core eudicots and the monocots rice and maize suggest a conserved function for *AG *homologs in angiosperms. At the same time gene phylogenies reveal a complex history of gene duplications and repeated subfunctionalization of paralogs.

**Results:**

*EScaAG1 *and *EScaAG2*, duplicate *AG *homologs in the basal eudicot *Eschscholzia californica *show a high degree of similarity in sequence and expression, although *EScaAG2 *expression is lower than *EScaAG1 *expression. Functional studies employing virus-induced gene silencing (VIGS) demonstrate that knock down of *EScaAG1 *and *2 *function leads to homeotic conversion of stamens into petaloid structures and defects in floral meristem termination. However, carpels are transformed into petaloid organs rather than sepaloid structures. We also show that a reduction of *EScaAG1 *and *EScaAG2 *expression leads to significantly increased expression of a subset of floral homeotic B genes.

**Conclusions:**

This work presents expression and functional analysis of the two basal eudicot *AG *homologs. The reduction of *EScaAG1 *and *2 *functions results in the change of stamen to petal identity and a transformation of the central whorl organ identity from carpel into petal identity. Petal identity requires the presence of the floral homeotic B function and our results show that the expression of a subset of B function genes extends into the central whorl when the C function is reduced. We propose a model for the evolution of B function regulation by C function suggesting that the mode of B function gene regulation found in *Eschscholzia *is ancestral and the C-independent regulation as found in *Arabidopsis *is evolutionarily derived.

## Background

Flowers are complex structures composed of vegetative and reproductive organs that are arranged in concentric whorls in most angiosperms. The vegetative floral organs, the sepals and the petals, develop in the outer whorls while the inner whorls are composed of the pollen-bearing stamens and in the center carpels enclose the ovules. The carpels are the last organs formed in the flower and the floral meristem is consumed in the process of carpel development [1]. As described by the ABCDE model, floral homeotic transcription factors act in a combinatorial fashion to determine the organ identity primordia for the four distinct whorls: A + E class genes specify sepal identity; A + B + E class genes act together to determine petal identity; B + C + E class genes specify stamen identity; C + E class genes together define carpel identity, and C + D + E class genes specify ovule identity [2,3]. Most of these homeotic functions are performed by members of the MADS-box gene transcription factor family. *AGAMOUS (AG), a *C class gene in *Arabidopsis *is necessary for specification and development of stamen and carpals, and floral meristem determinacy [4]. The flowers of the strong *ag-1 *mutant shows complete homeotic conversions of stamens into petals and carpels into sepals and a recurrence of these perianth organs in a irregular phyllotaxy [5].

**Figure 1:**
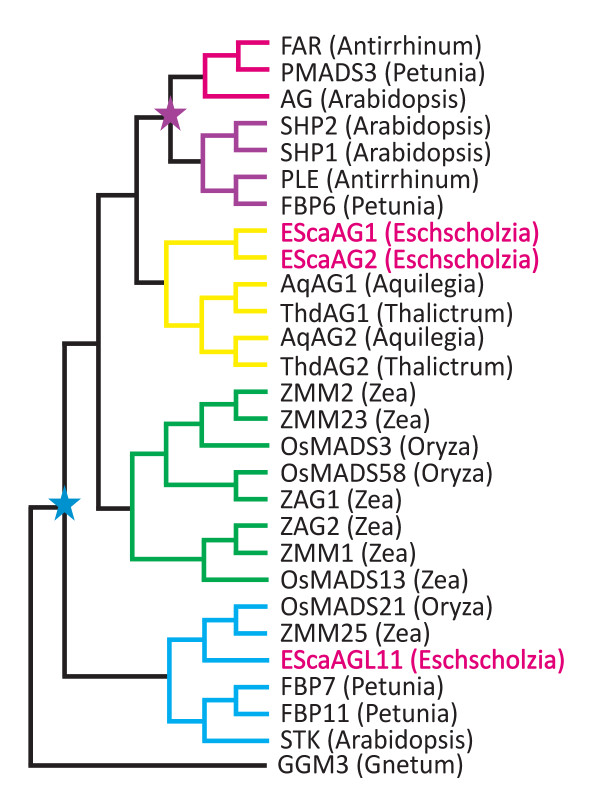
**Simplified phylogeny indicating duplication events of the AG lineage in angiosperms based on Zahn *et al*., 2006 **[18]. Red branches denote euAG lineage genes, purple branches the PLE lineage genes, yellow branches symbolize the basal eudicot lineage, green branches denote the monocot C class genes and blue branches denote D class genes. *GGM3 *represents the gymnosperm lineage of *AG *homologs. The California poppy genes are marked in red letters. The blue star symbolizes the C/D duplication event and the purple star indicates the EuAG/PLE duplication.

Members of the *AG *subfamily of MADS box genes have been identified in all major clades of seed plants but not in more basal, seed-free lineages indicating that the *AG *clade originated around 300 to 400 million years ago in the common ancestor of gymnosperms and angiosperms. In gymnosperm species, *AG *orthologs were found to be expressed in male and female reproductive cones, which is reminiscent of the angiosperm expression in stamens and carpels [6-8]. Gene family phylogenies reveal several duplication events within *AG *clade of MADS box genes (Figure 1[9,10]). The first duplication event at the base of the angiosperm lineage led to the origins of the *SEEDSTICK *and *AG *clades including ovule specific D class genes and the carpel and stamen specifying C class genes, respectively [10]. A more recent duplication in the C-lineage gave rise to the *PLENA *clade and *euAG *clade, the former containing the Arabidopsis *SHATTERPROOF1 *and *2 *genes *(SHP1 *and *2*), the latter *AG*. This duplication occurred after the ranunculids (basal eudicots in the order Ranunculales) diverged from the lineage leading to the core eudicots [9,11].

The Arabidopsis members of the *PLENA *clade, *SHP1 *and *2 *are required for dehiscence zone differentiation in the fruit and consequently for pod shattering [12,13]. Interestingly, *PLENA *itself, a gene in *Antirrhinum majus*, is functionally more similar to *AG *than *SHP1 *and *2*, and *FARINELLI (FAR)*, the *Antirrhinum AG *ortholog is required for pollen development. Both *FAR *and *PLENA *are necessary for floral meristem determinacy in *Antirrhinum *[14,15].

Gene duplications and subfunctionalization have also occurred in C-lineage of monocots, but independently of the eudicot duplications (Figure 1). *ZAG1 *from maize is required for floral meristem determinacy and *ZMM2 *is involved in stamen and carpel identity [16]. The rice homologs *OSMADS3 *and *OSMADS58 *share common functions, but also show a degree of subfunctionalization. While *OSMADS3 *plays a major role in stamen and a minor role in carpel identity, *OSMADS58 *has a strong influence on carpel identity and floral meristem determination [17]. Independent duplications of *AG *homologs have been inferred for other flowering plant lineages, but functional analyses of duplicated AG homologs are sorely lacking outside of model core eudicot and grass species.

Here we report functional data of the *AG *homologs of the basal eudicot *Eschscholzia californica *(California poppy, Papaveraceae) that belongs to Ranunculales, a basal eudicot order. Basal eudicots are a sister grade leading to the more diverse core eudicot clade. Investigation of species in this grade can shed light on the divergence of monocots and eudicots and events that may have promoted diversification within the core eudicots.

Two *AG *homologs, *EScaAG1 *and *EScaAG2*, and a D lineage homolog, *EScaAGL11 *, have been identified in *E. californica*. *EScaAG1 *and *EScaAG2 *show similar expression patterns, but *EScaAG1 *is expressed at a much higher level than *EScaAG*2 [18]. The expression patterns of both genes resembles that of *AGAMOUS *(*AG*) in *Arabidopsis *except that the Eschscholzia poppy *AG *orthologs are expressed earlier in the floral meristem [18,19].

This work presents an experimental investigation of the *EScaAG1 *and *EScaAG2 *gene function employing VIGS to manipulate transcript concentrations. We map the expression of both genes in more detail than previously published and demonstrate that the down regulation of C function genes in *E. californica *leads to an induction of some floral homeotic B genes in the fourth floral whorl.

## Results

### *EScaAG1 *and *EScaAG2 *are very similar in sequence and expressed differentially

The two *AG *homologues of *E. californica, EScaAG1 *and *EScaAG2*, share 66.6% and 61.1% amino acid sequence identity to *AG *of *Arabidopsis*, respectively. These paralogs are very similar throughout the open reading frame and in the 5'untranslated region (UTR) with 75% identity at the nucleotide level and about 81.7% at the amino acid level (Additional file 1). When the two paralogues are compared along their UTR and open reading frame, the *EScaAG*2 nucleotide sequence shows a 45 bp insertion and 14 bp deletion in the 5' UTR and a 10 bp deletion in the 3' part of coding region of *EScaAG1 *(data not shown).

**Figure 2:**
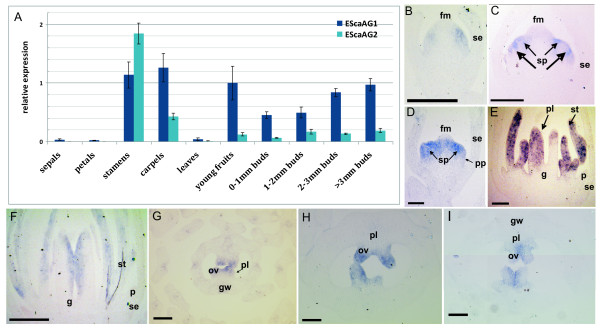
**Expression analysis of *EScaAG1 *and *EScaAG2 *in flowers of untreated plants shown by quantitative RT-PCR and *in situ *hybridization**. **(A) **Q-PCR based relative expression analysis of *EScaAG1 *and *EScaAG2 *in *E. californica. Actin *and *GAPDH *were used as reference genes. **(B) **to **(I) ***in situ *hybridization pattern of the *EScaAG *genes using an *EScaAG1 *probe. **(B) **Longitudinal section of a bud in stage 2. **(C) **Longitudinal sections of a bud in stage 3. **(D) **Longitudinal sections of a bud in stage 4. **(E) **Longitudinal section of a bud in late stage 6. **(F) **Longitudinal section of a bud in stage 7. **(G) **Transverse section of a bud in stage 7. **(H) **Transverse section of a bud in stage 8. **(I) **Transverse section of a bud in stage 9. All scale bars = 100 μm, abbreviations: fm, floral meristem; g, gynoecium; gw, gynoecium wall; ov, ovule; p, petal; pl, placenta; pp, petal primordium; se, sepal; sp, stamen primordium; st, stamen.

Quantitative Reverse Transcriptase (RT)-PCR was carried out on cDNA derived from floral organs at anthesis, young fruits, leaves, and buds of different developmental stages to learn more about the differential expression of *EScaAG1 *and *EScaAG2 *(Figure 2A). Both genes are expressed in the reproductive organs of the flower, in young fruits and in all tested stages of flower development. *EScaAG1 *and *EScaAG2 *are expressed in sepals, petals, and leaves at extremely low levels. *EScaAG1 *is highly expressed in stamens, carpels, young fruits and later stages of flower development. *EScaAG2 *is generally expressed at a lower level than *EScaAG1 *with the exception of stamen, where its expression is about 1.5 × higher than that of *EScaAG1*. In young fruits and during bud development, *EScaAG2 *transcript abundance is very low in comparison to *EScaAG1*.

The spatial expression patterns of *EScaAG1 *and *2 *were additionally analyzed through *in situ *hybridizations to obtain a more detailed picture of the expression domains. However, as the open reading frames and UTR's of *EScAG1 *and *EScaAG2 *are highly similar, we were unable to generate probes that could discriminate between both genes. As a consequence, *in situ *hybridization patterns were nearly identical for these genes. The only difference between the *in situ *hybridization patterns was a much lower level of expression for *EScaAG2 *(data not shown). In the following section, we refer to the composite expression of *EScaAG1 *and *EScaAG2 *as *EScaAG1/*2 expression patterns.

*EScaAG1/2 *gene expression was first observed in the stage 2 bud before the gynoecium initiates and was visible as lateral domains in a few cells in the floral meristem where later the stamen primordia are initiated (Figure 2B). In a stage 4 bud, the expression expands uniformly in the floral meristem but is excluded from the central primordium where later the gynoecium arises (Figure 2C). By late stage 4, *EScaAG1/2 *expression becomes restricted to the boundaries between the stamen anlagen with weak expression at the tip in the floral meristem just before gynoecium initiates (Figure 2D). In stage 6, strong expression is found in the region adjacent to the placenta, the apical part of the medial carpel wall and in the stamens (Figure 2E). Later in late stage 6, *EScaAG1/2 *expression is restricted to the adaxial side of the gynoecium and in the stamens (Figure 2F). In transverse sections of the developing flower bud, *EScaAG *expression is confined to the apical part of the ovules but not in the placenta. In later stages of ovule development, the *EScaAG1/2 *expression is stronger on the adaxial than on the abaxial side (Figure 2G, H, I). In summary, *EScaAG1/2 *genes are expressed during floral meristem initiation at stage 2, during early development of stamen and carpel primordia and later in the developing stamens and ovules.

### *EScaAG1 *and *EScaAG2 *confer stamen identity

**Figure 3:**
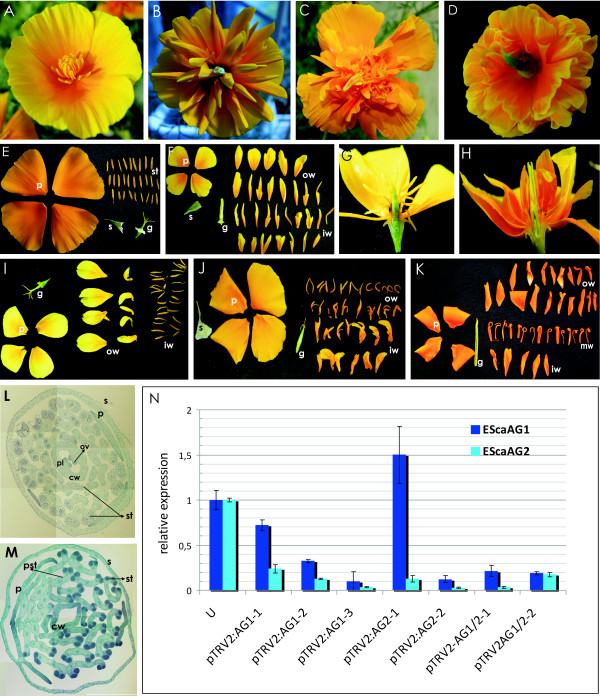
**Phenotypes of plants treated with pTRV1 and pTRV2-EScaAG1, pTRV2-EScaAG2, or pTRV2-EScaAG1/2 and expression analysis of the VIGS treated plants**. **(A) **Wild type phenotype of an *E. californica *flower treated with pTRV2-E. **(B) **Phenotype of an *EScaAG1 *VIGS treated plant showing full homeotic conversions of stamens into petals. **(C) **Phenotype of an *EScaAG2 *VIGS treated plant showing full homeotic conversions of stamens and carpels into petal-like structures. **(D) **Phenotype of a flower silenced for *EScaAG1/2 *showing homeotic conversions of stamens into petals. **(E) **A mock treated plant with disassembled floral organs. **(F) **Disassembled flower of a plant treated with pTRV2-EScaAG1/2 showing homeotic conversions of stamens into petals. The same phenotype was also achieved with plants silenced for *EScaAG1 *or *EScaAG2 *individually. **(G) **Transverse hand section of a flower from a mock treated plant. **(H) **Transverse hand section of a flower from a plant silenced for *EScaAG1/2 *showing homeotic conversions of stamens into petals. **(I) **Disassembled flower of a plant silenced for *EScaAG1 *showing partial homeotic conversions of only the outer whorl stamens. **(J) **Disassembled flower of a plant treated with pTRV2-EScaAG2 showing that the inner whorl of stamens is converted into petal-like structures. **(K) **Disassembled flower of a plant treated with pTRV2-EScaAG1/2 exhibiting homeotic conversions of the innermost and outermost stamens whorls into petals while the middle whorls show mild deviation from wild type stamens while the center whorl stamens remain more stamen-like. **(L) **Transverse section of a flower of an untreated plant. **(M) **Transverse section of a flower from an *EScaAG1 *VIGS treated plant showing homeotic conversion of stamens into petals, petal-stamen mosaic structures, malformed stamens and a gynoecium lacking tissue differentiation, ovules, and placenta. **(N) **Real-Time PCR analysis of the first bud of individual *E. californica *plants treated with VIGS and untreated (U). Plants were treated with pTRV2-EScaAG1 are abbreviated as VIGS AG1, plants treated with pTRV2-EScaAG2 as VIGS AG2. Numbers below indicated individual plants and the relative expression level of *EScaAG1 *and *EScaAG2 *in untreated plants was set to 1. Abbreviations: cw, carpel wall; ov, ovule; p, petal; pl, placenta; se, sepal; pst, petaloid stamens; st, stamen.

Virus induced gene silencing (VIGS ) was employed to investigate the functions of *EScaAG1 *and *EScaAG2 *during flower development. This method allows transient down-regulation of gene expression via modified plant viruses, in our case the Tobacco Rattle Virus (TRV). The *E. californica *flower is composed of a single sepal occupying the first floral whorl, two whorls of four petals and a varying number of stamen whorls ranging from four to eight. The inner floral whorl produces a bicarpellate gynoecium (Figure 3A) [20]. Overall, the phenotypic effects of the *EScaAG1 *and *EScaAG2 *VIGS were restricted to flowers. Treatment plants exhibited a loss of stamen identity, homeotic conversion of stamens into petals, and a loss of carpel characteristics. Additionally, *EScaAG1 *and *2 *VIGS results in a loss of floral meristem termination. None of the analyzed pTRV2-E (mock treatment, treated with the empty pTRV2 vector) treated or untreated plants showed homeotic conversions or signs of loss of floral meristem termination, and the vegetative habit also did not show any deviations from untreated plants (Table 1 and [21]).

**Table 1:** Overview of the observed phenotypes of EScaAG VIGS in California poppy

	Phenotypes observed	pTRV1/pTRV2-E	pTRV1/pTRV2-EScaAG1	pTRV1/pTRV2-EScaAG2	pTRV1/pTRV2-EScaAG1+2
**1**	No. of inoculated plants	12	120	120	120

**2**	No. of analyzed flowers	36	239	209	261

**3**	No. of flowers showing phenotype in the third and fourth whorls	0	122 (51.0%)	118 (56.4%)	174(66.6%)

**3.1**	No. of flowers with homeotic conversions in the stamens	0	67 (54.9%)	53 (44.9%)	113 (64.9%)

3.1.1	No. of flowers with transformation of all the stamens into petals	0	3 (4.4%)	8 (15%)	17 (15%)

3.1.2	No. of flowers showing only outer stamen whorls converted into petaloid organs	0	64 (95.5%)	0	0

3.1.3	No. of flowers showing only inner stamen whorls converted into petaloid organs	0	0	45 (84.9%)	0

3.1.4	No. of flowers showing only outer and inner stamen whorls converted into petaloid organs	0	0	0	96 (84.9%)

**3.2**	**No. of flowers with alterations in the carpels**	**0**	**27 (22.1%)**	**31 (26.2%)**	**40 (22.9%)**

3.2.1	No. of flowers with flattened green gynoecium	0	23 (85.1%)	26 (83.8%)	32 (80%)

3.2.2	No. of flowers with an orange pigmented gynoecium	0	4 (17.3%)	5 (16.1%)	8 (20%)

**3.3**	**No. of flowers showing defects in the floral meristem termination**	**0**	**62 (50.8%)**	**80 (67.7%)**	**110 (63.2%)**

In total, 120 plants were infected with pTRV2-*EScaAG*1, 120 plants with pTRV2-*EScaAG*2, another 120 plants were inoculated with pTRV2-*EScaAG1/2*, and 12 plants were infected with pTRV2-E as a mock control. The first three flowers of each plant were analyzed because the frequency of phenotype decreases in the later formed flowers [21]. The phenotype scores for each treatment are summarized in Table 1: 239 flowers of plants infected with pTRV2-*EScaAG1 *were analyzed, of which 122 flowers (51.0%) showed homeotic conversion in the third and fourth whorl floral organs. Of these 122 flowers, 4.5% showed homeotic conversion of all stamens into petal-like organs (Figure 3B). A total of 209 flowers of plants infected with pTRV2-*EScaAG*2 were observed, and of these 118 flowers (56.4%) showed homeotic transformation of stamens and carpels. Of all flowers developing a silencing related phenotype, 15% exhibited complete homeotic transformation of all stamens into petal-like organs (Figure 3C). Of the 261 flowers of plants infected with pTRV2-*EScaAG*1/AG2, 174 flowers (66.6%) showed homeotic transformation of stamens and carpels and 15% of the latter exhibited complete homeotic transformation of all stamens into petal-like organs (Figure 3D-H, Table 1).

Interestingly, *EScaAG1 *and *2 *VIGS-treated plants exhibited conversion of stamen to petaloid organs in different stamen whorls (Table 1). Focusing on plants infected with pTRV2-EScaAG1, 64 flowers (95.5% of the flowers with homeotic conversion in the third whorl) showed partial homeotic transformation of only the outer whorls of stamens into petaloid organs (Figure 3I), while the inner stamen whorls maintained a wild type appearance. In contrast, 45 flowers (84.9% of the flowers with homeotic conversions in the third whorl), from plants infected with pTRV2-EScaAG2 showed homeotic conversion of only the inner stamen whorls to petaloid organs (Figure 3J). Plants infected with pTRV2-EScaAG1/2 exhibited composite phenotypes: 96 flowers (84.9% of the flower with homeotic conversion in the third whorl) exhibited partial homeotic conversion of outermost and innermost whorls while retaining wild type stamen morphology in the central stamen whorls (Figure 3K). Homeotic transformations of stamens into petaloid organs occurred in various degrees as we observed phenotypes ranging from complete petal-like organs (Figure 3F) to mosaic staminoid-petaloid structures (Figure 3K)..

Histological transverse sections of *EScaAG1 *and *2 *VIGS-treated plants reveal further details of the homeotic conversions of stamens and gynoecia (Figure 3L, M). In comparison to pTRV2-E treated plants (Figure 3M), the connective of the stamens in the silenced plants is elongated when compared to untreated plants and the theca contain three pollen sacs in a few cases rather than two as seen in untreated plants. The number of vascular bundles in the connective is also increased from one in untreated to five in stamens of VIGS treated plants. Additionally, the gynoecium in the center of the flower of VIGS-treated plants is composed of two fused parts reminiscent of petals. A solid ovary wall is missing in the VIGS-treated plants as well as lateral differentiation of the ovary wall, such as a placenta or ovules (Figure 3L, M).

The strength of the observed phenotypes was correlated with the degree of reduction in *EScaAG1 *and *2 *transcript levels as measured by Q-PCR. The first floral bud (size 1 to 3 mm in diameter) of randomly selected plants treated with the *EScaAG1, EScaAG2, and EScaAG1/2 *VIGS vectors was collected and correlated with the phenotype of the next formed flower. In 99% of the cases (n = 414) we observed that when the secondarily formed flower showed a phenotype, the first flower exhibited a phenotype as well. This consistent pattern allowed us to predict the phenotype of the first bud used for quantitative RT-PCR based on the second flower's phenotype (see also [21,22]). The changes in *EScaAG1 *and *EScaAG2 *expression in the first buds (1 to 3 mm bud diameter) of individual VIGS treated plants are documented in Figure 3N. Targeted silencing of individual *EScaAG *genes was not achieved, suggesting that the overlap in observed phenotypes result from a reduction of expression of both *AG *paralogs. Irrespective of the silencing vector used, *EScaAG1 *expression was generally reduced from 70% to 10% of its wild type expression and *EScaAG2 *expression was reduced from 25% to less than 5%. The use of the pTRV2-EScaAG1/2 vector resulted in similar reductions in expression levels for both genes. While six plants show silencing of both, *EScaAG1 *and *EScaAG2*, one plant (pTRV2:AG2-1) treated with *EScaAG2*-targeted VIGS exhibited reduction of *EScaAG2 *expression but increased *EScaAG1 *expression relative to untreated plants, demonstrating the variability of VIGS experiments. However, we were able to show a significant reduction of expression in six of seven randomly analyzed buds from individual plants.

### VIGS of C-function genes results in homeotic conversions of carpels into petal-like organs

**Figure 4:**
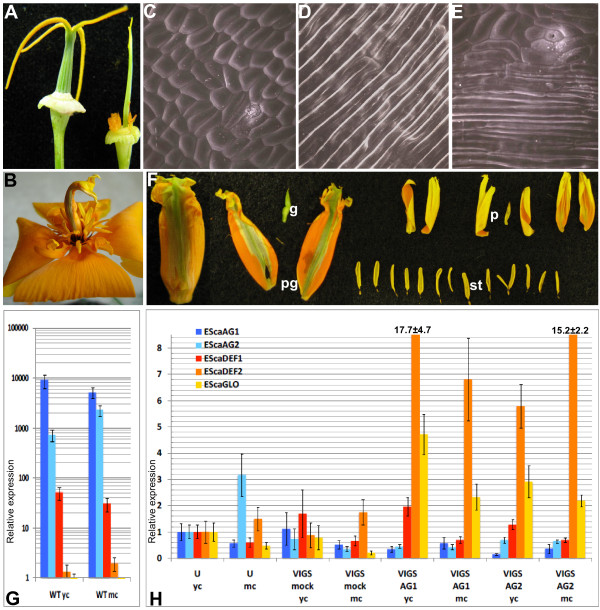
**Carpel whorl phenotype of *EScaAG *silenced plants and expression analysis of floral homeotic genes**. **(A) **Gynoecium of an untreated plant (left) and of a plant silenced for *EScaAG2 *(right) **(B) **Flat orange gynoecium without ovules of a plant treated with pTRV2-*EScaAG1*. **(C) **Scanning electron micrograph (SEM) of the wild type gynoecium surface structure. **(D) **SEM of a wild type petal surface structure. **(E) **SEM of the central floral whorl organ of a plant treated with pTRV2-*EScaAG1 *showing a mix of petal and gynoecium surface structures. **(F) **The central whorl floral organ (pg, for petaloid gynoecium) of a plant treated with pTRV2-*EScaAG2 *showing petaloid and carpeloid features as well a lack of ovules. This organ encloses an ectopic flower consisting of a remnant gynoecium (g), petals (p) and stamens (st). **(G) **Relative expression of class B and C genes in young carpels before anthesis and mature carpels at anthesis of untreated plants, **(H) **Real-Time RT-PCR expression analysis of *EScaAG1, EScaAG2, EScaDEF1, EScaDEF2*, and *EScaGLO *in the gynoecia of VIGS treated plants. Abbreviations used in (G) and (H): yc, young carpel before anthesis; mc, mature carpel at anthesis; u, untreated plants; pTRV2-E, plants treated with pTRV1 and pTRV2-E; pTRV2-AG1, plants silenced for *EScaAG1*; pTRV2-AG2, plants silenced for *EScaAG2*; pTRV2-AG1/2, plants silenced for *EScaAG1 *and *EScaAG2*.

In addition to homeotic conversions of stamens into petal-like structures in plants infected with pTRV2-*EScaAG1 *and pTRV2-EScaAG2, we observed changes to the gynoecium morphology. The gynoecia of untreated plants develop as round green cylinders and consist of two fused carpels. This cylinder-like structure was disturbed in *EScaAG1 *and *EScaAG2 *VIGS-treated plants and the gynoecia of the VIGS treated plants were transformed either into (i) flattened green structures lacking ovules in some cases (Figure 4A Table 1) or (ii) flattened organs showing petal characteristics such as orange pigmentation and petal-like epidermal surface structure (Figure 4B Table 1). The latter was empty (Figure 4A, B) or contained additional floral organs (Figure 4F Table 1).

In order to determine whether the petal-like pigmentation of the gynoecium was associated with a change in cell surface morphology we conducted Scanning Electron Microscopy (SEM) analysis of the carpel whorl. In the wild type, the carpel surface is composed of small compact cells interrupted by stomatal cells (Figure 4C) and the petal surface is composed of long and narrow cells arranged in a parallel manner (Figure 3D) [20]. SEM micrographs of an orange-pigmented gynoecium reveal a mosaic pattern of tubular petal-like cells next to small compact cells typical for a carpel surface scattered with stomata (Figure 3E). This indicates that the gynoecia of *EScaAG1 *and *EScaAG2 *VIGS treated plants not only show a partially petal-like pigmentation but have also acquired petal-like cell surface characteristics, supporting the hypothesis that these gynoecia are partially transformed into petal-like organs.

Treating poppy plants with *EScaAG1 *and *2 *VIGS not only resulted in the loss of stamen and carpel characteristics but also in the addition of petal organ identity to the carpel whorl. We tested the hypothesisis that the expression domains of floral homeotic B genes was extended to the central gynoecium whorl in *EScaAG1 *and *2 *VIGS treated plants using real-time PCR to assess expression of the three poppy floral homeotic B class genes *EScaDEF1*, *EScaDEF2*, and *EScaGLO *at anthesis and pre-anthesis (Figure 4G, H). B and C gene expression in untreated gynoecia was also characterized (Figure 4D). As expected *EScaAG1 *as well as *EScaAG2 *were expressed in gynoecia before and at anthesis. Surprisingly, the class B gene ortholog, *EScaDEF1 *was expressed in gynoecia at a comparatively high level, although expression levels of two other B-class genes *EScaDEF2 *and *EScaGLO *were hardly detectable. Next, the expression of class B and C genes was recorded in the gynoecia of VIGS treated plants (Figure 4G). The relative expression of all analyzed genes was normalized by setting levels to one in gynoecia of untreated plants before anthesis. In the gynoecia of VIGS treated plants (Figure 4H), expression of *EScaAG1 *was reduced to 50% and even 20% in the gynoecia of VIGS treated plants and expression of *EScaAG2 *was reduced in most gynoecia as well. VIGS treatments had no impact on *EScaDEF1 *expression in the gynoecia. However, the expression of *EScaDEF2 *was drastically increased between 5.8-fold and 17.7-fold relative to expression in untreated gynoecia. Transcript abundance of *EScaGLO*, also increased significantly upon silencing of C function genes by 2.2 to 5.7 times in the *EScaAG1 *and *EScaAG2 *VIGS treated plants. These expression analyses indicate that in central whorl organs with reduced expression of C function genes, two B function genes *EScaDEF2 *and *EScaGLO *were expressed at significantly higher level in *EScaAG1 *and *EScaAG2 *VIGS treated than in untreated or mock treated plants.

For the *Arabidopsis *B proteins APETALA3 (AP3) and PISTILLATA (PI) it was shown that their homeotic function requires the formation of AP3-PI heterodimers [23]. EScaDEF2 is an AP3 homolog while EScaGLO is the PI homolog [24] and simultaneous upregulation of the AP3 and PI orthologs in poppy suggests that they might form heterodimers in the central whorl of C function silenced flowers and cause the observed homeotic gynoecium-petal conversions.

### *EScaAG1 *and *EScaAG2 *are involved in the regulation of floral meristem termination

The flowers of the plants treated with *EScaAG1 *and *EScaAG2 *VIGS showed not only homeotic conversions of stamens into petaloid organs, petal-like features in the central whorl, and a reduction in ovule number, but also signs of prolonged floral meristem activity. All treated plants showed increases in floral organ number in the stamen and central whorls. Moreover, flowers exhibiting a strong silencing phenotype showed ectopic structure enclosed inside the gynoecium whorl ranging from carpel like leaves to additional gynoecia and ectopic flowers (Figure 4F).

Interestingly, we observed a significant increase in stamen number in the weaker floral phenotypes characterized by no obvious homeotic organ conversions (Table 2). Untreated plants produced 26.2 stamens per flower on average, *EScaAG1 *VIGS-treated plants without any homeotic conversions developed 29 stamens per flower, *EScaAG2 *VIGS-treated produced 28.2, and plants treated simultaneously with *EScaAG1/2 *produced 28.6 stamens on average. This suggests that whereas a mild reduction in *EScaAG1 *and *EScaAG2 *expression may not affect floral organ identity any reduction in expression can induce an increase in stamen number.

**Table 2:** Stamen numbers of in EScaAG1, EScaAG2, and EScaAG1/2 VIGS treated plants

	Untreated/pTRV1 and pTRV2-E treated	EScaAG1 VIGS treated	EScaAG2 VIGS treated	EScaAG1/2 VIGS treated
**No. of flowers analyzed**	28	244	242	333

**No. of flowers without homeotic conversions**	28	93	123	92

**Average no. of stamens in flowers without homeotic conversions**	26.2 ± 1.9	29* ± 4.1	28.2* ± 2.9	28.6* ± 4.5

* Significant change to untreated control plants (ANOVA test)

## Discussion

This study is the first functional analysis of floral homeotic C function genes in a basal eudicot. We employed VIGS to transiently down-regulate *EScaAG1 *and *EScaAG2 *in *E. californica *and observed homeotic conversions of stamens into petals, reduced floral meristem termination, and transformation of the gynoecium into petal-like structures. *EScaAG2 *is expressed at lower levels (also observed by [18]) but despite the reduced expression of *EScaAG*2, molecular evolutionary analyses failed to detect evidence of reduced evolutionary constraint (see below).

The two *AG *paralogs of *E. californica*, *EScaAG1 *and *EScaAG*2 are quite similar on both protein and nucleotide level including the 5'UTR region indicating that they are duplicates. Generally it is hypothesized, that duplicated genes will not persist over evolutionary time unless sub-, or neofunctionalization results in functional divergence [25-27]. *EScaAG1 *and *EScaAG2 *share about 81.7% sequence similarity in the open reading frame and are 75.5% identical when the 5'UTR is included. The origin of these paralogs may be associated with an ancient whole genome duplication event that has been inferred on the lineage leading to *Eschscholzia *[28]. Using a penalized likelihood approach [29] we estimated an age of 51 million years for the *EScaAG *duplication. This divergence time was obtained using a maximum likelihood tree for the *AG *subfamily [30] calibrated with taxon ages reported in [31].

No evidence of reduced constraint on *EScaAG2 *was inferred from analysis of the ratio of nonsynonymous to synonymous nucleotide substitutions on the branch leading to *EScaAG2 *[30]. A recent shift in constraint on *EScaAG2 *may not be detectable [32], but the molecular evolutionary analyses indicate that both *EScaAG2 *and *EScaAG1 *have been evolving under selective constraint for much of the approximately 50 million years since duplication. These results suggest that both *EScaAG1 *and *2 *have been selectively maintained in the lineage leading to *E. californica*.

Gymnosperm and angiosperm *AG *homologs are highly conserved but gene duplications have spurred functional diversification. The observation that knocking down *EScaAG1 *and *2 *individually results in overlapping phenotypes can be explained by two alternative scenarios. First, the two poppy *AG *paralogs may be working redundantly in the specification of floral organ identity and floral meristem determinacy. Alternatively, the VIGS method may not be able to individually silence paralogs with highly similar sequences. Our results are not fully consistent with either of these interpretations. Expression analyses of single knock down VIGS plants showed that transcript abundance of both genes was decreased, but *EscaAG2 *was silenced more strongly than *EscaAG1*. With respect to the first scenario, the selective maintenance of fully redundant genes over 50 million years is highly unlikely. Full knockouts (vs. knock downs) for each paralog may be required to reveal subtle functional divergence.

Differences in expression between *EScaAG1 *and *EScaAG2 *(Figure 2A) and deviations in spatial distribution of the homeotic conversions of stamens into petals (Figure 3I-K) hint at some degree of subfunctionalization. However, we were not able to relate the distinct phenotypes of only outer stamen whorl homeotic conversions in the case of *EScaAG1 *VIGS and only inner stamen whorl conversion in the *EScaAG2-*silenced flowers to the expression *EScaAG1 *and *EScaAG2 *expression data. In almost all analyzed floral buds we have simultaneous down-regulation of both genes with always a higher residual *EScaAG1 *expression than *EScaAG2 *expression, suggesting that subtle spatial expression difference at a very early developmental stage might play a role which we were not able to detect with our expression analysis. A less transient approach such as stable transformation with hairpin RNA constructs that would be able to silence *EScaAG1 *and *EScaAG2 *expression individually is required to rigorously characterize functional domains for *EScaAG1 *and *2*, and test the subfunctionalization hypothesis.

**Figure 5:**
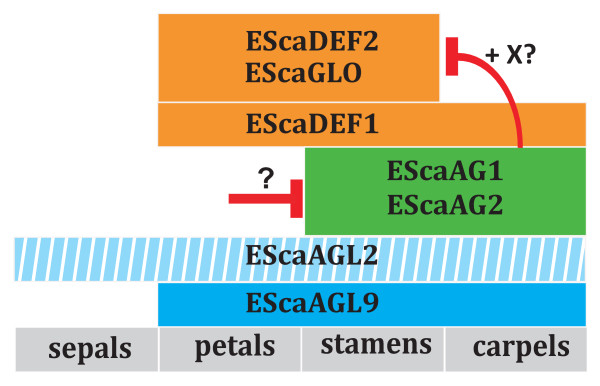
**Hypothesis on the regulation of class C dependent B gene expression in *E. californica***. This modified BCE model of *E. californica *floral organ identity specification includes the B class genes (orange boxes) *EScaDEF1*, *EScaDEF2*, and *EScaGLO *that are supposed to be expressed in second and third (stamen) whorl. *E*xpression of *EScaDEF1 *is also found in the central whorl. Two class C genes (green boxes) are expressed in the stamen and central whorl, and the two class E genes *EScaAGL2 *and *EScaAGL9 *(blue boxes) are expressed in all whorls except for the sepal whorl for *EScaAGL9 *[43] while no information is available on the expression domain of *EScaAGL2*. Red bars indicate repression of gene expression. It remains unclear by which mechanism *EScaAG1 *and *EScaAG2 *expression is restricted to the reproductive whorls of the flower and if repression of *EScaDEF2 *and *EScaGLO *by the two class C genes is direct or mediated by a co-factor. An A function has not yet been described in California poppy.

Another characteristic of the *EScaAG1 *and *2 *VIGS phenotype is the loss of carpel organ identity. The most common phenotype observations were flattened green gynoecia or flat petaloid gynoecia showing an orange pigmentation and cell surface structure typical for petals (Figure 4A-E, Additional file 2.). The latter finding indicates that in *E. californica*, homeotic conversions of gynoecia into petaloid structures can occur when the C function is missing. This homeotic conversion coincides with the expansion of the expression domains of two class B genes, *EScaDEF2 *and *EScaGLO*, into the central floral whorl of *EScaAG1 *and *EScaAG2 *VIGS treated plants. The third B class gene, *EScaDEF1 *is also expressed in the gynoecia of untreated plants and expression levels are unaffected by reduction of C class gene expression in VIGS treated plants (Figure 5). These findings suggest that *EScaDEF1 *expression is independent of class C gene expression while *EScaDEF2 *and *EScaGLO *are negatively regulated by class C genes in the central floral whorl.

Interestingly, *EScaDEF2 *and *EScaGLO *are expressed in parallel with the class C genes in the stamen whorl which indicates C independent expression of the two class B genes in stamen whorls in contrast to C dependent expression in the central floral whorl. Thus, a cofactor (X) restricted to the central whorl can be postulated to inhibit expression of *EScaDEF2 *and *EScaGLO *expression along with the C class proteins EScaAG1 and EScaAG2 (Figure 5).

This type of C-dependent regulation of B class genes is in contrast to the strong *Arabidopsis ag-3 *mutant, where full homeotic conversions of stamens into petals and carpels into sepals are observed. Even in the weaker *ag-4 *mutant, the carpel is not converted into a petal-like structure, but rather into a sepal [33]. Single or double mutants *shp1*/*shp2 *do not show any floral homeotic functions in *Arabidopsis*. Phenotypic effects are detectable only after fertilization [34]. In contrast, the *Antirrhinum ple-1/far *double mutant shows the type of floral homeotic conversions we observe in poppy: carpels are converted into petal-like structures and additional flower enclosed inside the fourth whorl unlike in the third whorl in Arabidopsis [15]. In the *Arabidopsis ag *mutant, the expression of the B function genes *AP3 *and *PI *in the fourth whorl is prevented by the action of *SUPERMAN *(*SUP*) [35] and carpels are converted to sepal-like organs [36]. Therefore, it seems that the regulation of B function genes is independent of C class gene function in *Arabidopsis*. However, at this point we cannot exclude the hypothesis that *AG *together with the closely related *SHP1 *and *SHP2 *genes work with *SUP *to repress B gene expression in the fourth whorl. In the *Antirrhinum ple-1/far *double mutant, an expansion of the B function expression domain towards the fourth whorl was observed as a result of a C function reduction. It was suggested that the putative *SUP *orthologs in *Antirrhinum, OCTANDRA *(*OCT*) requires *PLE *or *FAR *to exclude B function gene expression from the fourth whorl while *SUP *in *Arabidopsis *acts independently of *AG *[15].

Our analysis of the *EScaAG1 *and *EScaAG2 *VIGS flowers suggests that the regulation of poppy B function genes is more similar to *Antirrhinum *than to *Arabidopsis *because we also find an expansion of petal-like tissues specified by the B function in the fourth whorl. As postulated for *Antirrhinum*, the negative regulation of B function genes in the fourth whorl may involve the activation of an *E. californica SUP *ortholog. A poppy *SUP *ortholog could be positively regulated by *EScaAG1 *and *2 *or interact with these genes to restrict B function expression to the second and third whorl in wild type plants.

The fact that B gene expression is restricted by C function in *E. californica *as a representative of a basal eudicot lineage and *Antirrhinum*, a member of the asterid clade, in contrast to C independent regulation in *Arabidopsis *indicates that the former regulatory scenario might be ancestral. However, C function dependent regulation of B class genes has also not been reported in monocots such as rice. Down regulation of the rice *AG *homolog, *OsMADS58*, did not result in expansion of the expression domains for B class genes and carpel to lodicule transformation have not been observed in *osmads3 *mutants or *OsMADS58 *RNAi lines [17].

This suggests three possibilities for the evolution of class C dependent regulation of class B gene expression: (i) This type of regulation had evolved before the monocot and eudicot lineages diverged but was lost independently, in lineages leading to *Arabidopsis *and rice. (ii) The C-dependent regulation of B expression evolved once in the eudicots before the divergence of Ranunculales and was lost in the lineage leading to Arabidopsis after their split from the asterids. (iii) Class C genes were recruited twice independently, once in the lineage that led to *E. californica *after it diverged from the rest of the dicots and a second time in the lineage leading to *Antirrhinum *after its divergence from the lineage leading to *Arabidopsis*. Since class C floral homeotic mutants are not yet available from basal angiosperms or non-grass monocots all three of these scenarios are equally parsimonious.

As reported for *Arabidopsis ag *mutants, a reduction of *EScaAG1 *and *2 *function in *E. californica *leads to defects in floral meristem termination, albeit in a more complex pattern than observed in *Arabidopsis*. The stamen whorls of *EScaAG1 *and *2 *VIGS-treated plants are more numerous than in the control plants even if the phenotype is mild, for example, no homeotic conversions of reproductive organs (Figure 3F). These observations support inferences drawn from work on *A. thaliana *and *A. majus *where mild reductions in C-function affect floral meristem determinacy [37,38]. The morphogenesis of *E. californica *flowers differs from most core eudicots, for example, *Arabidopsis*, in that the innermost stamen whorls are still being formed when the central gynoecium is initiated. A ring of cells with meristematic activity around the gynoecium is maintained while the central floral meristem is consumed in the process of gynoecium initiation [20]. This suggests that a mild reduction in *EScaAG1 *and *2 *expression is sufficient for a prolonged meristem activity in this ring shaped meristem that produces additional stamen whorls in *EScaAG1 *and *2 *VIGS-treated flowers. Additionally, our results suggest that *EScaAG1 *and *2 *regulate the termination of meristem activity in *E. californica*. Regulation of meristematic activity was observed in the central floral meristem and the ring meristem that gives rise to stamen whorls independently of the ceasing central floral meristem activity (Table 2). The influence of *EScaAG1 *and *2 *VIGS on the stamen whorls is especially interesting as stamen numbers in wild type *E. californica *are phenotypically variable, ranging from 18 up to 34 stamens when individuals are grown under identical conditions and constant light [20]. Even slight differences in the timing and dose of *EScaAG1 *and *EScaAG2 *transcript abundance between plants could account for these stamen number variations in wild type plants. The number of stamens in *E. californica *generally coincides with the plant's stature: as has been reported for *Stellaria media *(chickweed) [39], healthier plants produce more stamens. Our analyses suggest that the number of stamens produced is dependent on the amount of *EScaAG1 *and *EScaAG2 *transcript in *E. californica *flowers. This might indicate a stature-dependent regulation of class C floral homeotic genes in the ring-like meristem. Moreover, a direct link could exist between floral homeotic gene action and male fecundity in natural populations.

This additional function of the class C genes in *E. californica *in the zone of meristematic activity around the gynoecium might represent a more general mode of function for class C genes in the large subgroup of angiosperms with several stamen whorls and often varying stamen numbers. The duration of class C genes activity in the meristems generating these reiterating stamen whorls might also determine stamen number in these species.

Our study on the function *EScaAG1 *and *EScaAG2 *in *E. californica *reveal that the VIGS method is suitable to analyze the evolution of gene regulation by enabling gene function analysis in non-model plants for which transgenic approaches are difficult to achieve. This work shows that gene function and the regulation of floral homeotic genes vary among plant lineages. Looking forward, the importance of VIGS for assessing gene function in non-model species will increase as advances in sequencing technologies result in full transcriptome and even genome sequences for an expanding number of species sampled across the plant tree of life. While sequence data will allow characterization of amino acid conservation and gene duplication events, functional studies in non-model species will be required to elucidate the evolution of regulatory networks influencing flowering time and floral form over angiosperm history.

## Materials and methods

### Expression analysis

#### Q-PCR

Q-PCR assays were performed on floral organs, leaves, young fruits, and buds of different developmental stages in wild type plants. For expression analysis of the *EScaAG1 *and *EScaAG2 *VIGS-treated plants, a single bud (1 to 2 mm in diameter) was examined. For the analysis of class C and B genes in VIGS-treated plants, single gynoecia of either buds of 5 to 8 mm diameter (young carpel) or from open flowers (mature carpel) were collected. All samples were analyzed in three technical replicates. One μg of total RNA was reverse transcribed into cDNA using random hexamer primers and the SuperScript III Kit (Invitrogen, Karlsruhe, Germany). A total of 5 μl of 1:50 diluted cDNA was used as a template. *DEF1 *and *Actin *primers were designed with the help of the UPL probe program (Roche, Mannheim, Germany); all other primers sets were designed with one intron spanning primer (primer details are in Additional file 3). Paralog specific primer pairs consist of forward primers spanning at least one intron and a reverse primer spanning the deletion part of *EScaAG1 *in 3' coding region were used to discriminate between *EScaAG1 *and *EScaAG2 *and the PCR product was sequenced to confirm primer specificity and the primer melting curves were analyzed. *Eschscholzia Actin2 *and *GAPDH *were used as reference genes. The Real-Time PCR reaction mix consisted of: 5 μl of cDNA (1:50 dilution), 10 μl of SYBR Green mix (Roche) and 0.8 to 1.2 pM primers. The UPL Real-Time PCR mix consisted of 5 μl of 1:50 diluted cDNA, 100 nM UPL probe (Roche, #132 for *EScaDEF1 *and #136 for *Actin*) and 0.04 pM of each primer. Real-Time PCR was performed using a Light Cycler 480 (Roche) with the following cycle conditions: initial heating of 95°C for 5 minutes, and 45 cycles of 10 s at 95°C, 10 s at 60°C and 10 s at 72°C. Cp values were analysed according to the Genorm manual and accurate normalization was carried out by geometric averaging of multiple internal control genes [40].

#### *In situ *hybridisation

Non-radioactive *in situ *hybridization followed essentially the protocol of [41]. The *EscaAG1 *and *EScaAG2 *coding regions were cloned into the pDrive vector (Qiagen, Hilde, Germany), the digoxigenin-labelled RNA probes were transcribed using SP6 polymerase (Roche) and subsequently hybridized to floral tissue sections.

#### Virus-induced gene silencing

A 395 bp fragment of *EScaAG1 *was amplified from the *EScaAG1 *coding region by using the primers VIGSEcAG1A to add a *BamH*I restriction site to the 5' end of the PCR product and EcAG1VIGS to add an *Xho*I restriction site to the 3' end (primer sequences reported in Additional file 3). The amplicon was digested with *BamH*I and *Xho*I and cloned into a similarly cut pTRV2 vector [42]. A 477 bp fragment of *EScaAG2 *was amplified from the *EScaAG2 *coding region by using the primers VIGSEcAG2A to add a *BamH*I restriction site to the 5' end of the PCR product and EcAG2VIGS to add an *Xho*I restriction site to the 3' end. The amplicon was digested with *BamH*I and *Xho*I and cloned into a similarly cut pTRV2. pTRV2-*EScaAG1/AG2 *was constructed by a 190 bp fragment of *EScaAG1 *was amplified from the *EScaAG1 *coding region by using the primers XbaVIGSEcAG1Bfw to add a *XbaI *restriction site to the 5' end of the PCR product and EcAG1VIGSXhorev to add a *Xho*I restriction site to the 3' end. The amplicon was digested with *XbaI *and *Xho*I. A 214 bp fragment of *EScaAG2 *was amplified from the *EScaAG2 *coding region by using the primers EcoVIGSEcAG2Afw to add an *EcoRI *restriction site to the 5' end of the PCR product and EcAG2VIGSXbarev to add an *XbaI *restriction site to the 3' end. The amplicon was digested with *EcoR*I and *Xba*I and was then ligated together with *the EScaAG1 *fragment into the *EcoRI *and *XhoI *cut pTRV2 vector producing the pTRV2-*EScaAG1/AG2 *plasmid. The vector inserts of the double construct were confirmed by restriction digestion and sequencing. The resulting plasmids were sequenced and transformed into *Agrobacterium tumefaciens *strain GV3101. The agro-inoculation was performed by injecting the *Agrobacterium *suspension into the shoot apical meristem as described by [22].

#### Scanning electron microscopy and histology

Gynoecia of *EScaAG1 *and *EScaAG2 *VIGS-treated and untreated plants were analyzed by Scanning Electron Microscopy [14] for changes in the cell surface structure. The gynoecia were incubated in 100% methanol for 10 minutes and subsequently for 10 minutes in 100% ethanol. Then they were kept overnight at room temperature in 100% ethanol and dried with a Critical Point Dryer, gold coated, and examined under the SEM (ISI-100B, International Scientific Instruments, Pleasanton, CA, USA). First formed buds of 1.6 to 2.5 mm in diameter were collected for histological analysis and stained with Safranin and Fast Green as described by [22].

## Abbreviations

AG: the floral homeotic C function gene *AGAMOUS of A. thaliana*; AP3: the floral homeotic B function gene *APETALA3 of A. thaliana*; EScaAG1: *E. californica *ortholog of *AG*; EScaAG2: *E. californica *ortholog of *AG*; EScaAGL 11: *E. californica *ortholog of the the ovule specific gene *SEEDSTICK *(formerly known as *AGL11*) of *A. thaliana*; EScaDEF1: *E. californica *ortholog of the *A. majus *floral homeotic B function gene *DEFICIENS*; EScaDEF2: *E. californica *ortholog of the *A. majus *floral homeotic B function gene *DEFICIENS *; EScaGLO: *E. californica *ortholog of the *A. majus *floral homeotic B function gene *GLOBOSA*; FAR: floral homeotic C function gene *FARINELLI *in A. majus; OCT: putative stamen and carpel boundary specifying gene *OCTANDRA *in *A. majus*; OSMADS3: floral homeotic C function gene of *O. sativa*; OSMADS58: floral homeotic C function gene of *O. sativa*; PI: the floral homeotic B function gene *PISTILLATA of A. thaliana*; PLE: the *A. majus *floral homeotic C function gene *PLENA*; Q RT-PCR: Quantitative Reverse Transcriptase polymerase chain reaction; SEM: Scanning electron microscopy; SHP: the *SHATTERPROOF *gene of *A. thaliana*; SUP: the stamen and carpel boundary specifying gene *SUPERMAN of A. thaliana*; TRV: Tobacco rattle virus; UPL: Universal probe library; UTR: Untranslated region; VIGS: Virus induced gene silencing; ZAG1: floral homeotic C function gene of Z. mays; ZMM2: floral homeotic C function gene Z. mays.

## Competing interests

The authors declare that they have no competing interests.

## Authors' contributions

AYL participated in the expression analysis and phenotype characterization. SL carried out the cloning work and participated in expression analysis and phenotype characterization. SO carried out and interpreted the *in situ *hybridizations. RE analyzed and interpreted the Real-Time PCR data. JLM performed sequence divergence time estimates and molecular evolutionary analyses and edited the manuscript. AB conceived of the study, participated in its design and coordination. AYL and AB wrote this manuscript. All authors read and commented on drafts of the manuscript and approved the final manuscript.

## Supplementary Material

Additional file 1 **Supplemental Figure 1: Alignment of the EScaAG1 and EScaAG2 protein sequences**. Amino acids identical between two paralogs are indicated by dots; dashes indicate deletion of five amino acids located in the C-terminal region of *EScaAG2*. Dissimilar residues are indicated by the respective amino acids. Click here for file

Additional file 2 **Supplemental Figure 2: Phenotypes observed in the gynoecium of *EScaAG *VIGS treated plants**. The Y-axis denotes the percentages of different carpel identity phenotypes obtained by VIGS (pTRV2-*EScaAG1*, n = 239; *EScaAG2*, n = 209, *EScaAG1/2*, n = 261 flowers). Differently treated VIGS plants are shown on the X-axis. The green color indicates the occurrence of flat green gynoecia; the orange color symbolizes flat orange gynoecia. Stripes indicate gynoecia enclosing ovules, plane color indicates a gynoecium lacking ovules, and the dotted pattern indicates additional organs enclosed by the gynoecium. Click here for file

Additional file 3 Supplemental Table 1: Sequences of primers used in this study. Click here for file
