# Supplementary figures and images for: Floral homeotic C function genes repress specific B function genes in the carpel whorl of the basal eudicot California poppy (Eschscholzia californica)
Source: EvoDevo. 2010 Dec 1;1:13. doi: 10.1186/2041-9139-1-13 (PMC3012024; doi:10.1186/2041-9139-1-13)

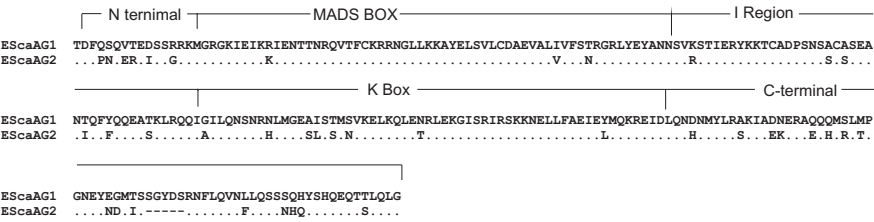

Supplement: Additional file 1 — Supplemental Figure 1: Alignment of the EScaAG1 and EScaAG2 protein sequences. Amino acids identical between two paralogs are indicated by dots; dashes indicate deletion of five amino acids located in the C-terminal region of EScaAG2. Dissimilar residues are indicated by the respective amino acids. [file 2041-9139-1-13-S1.PDF]

Suppl. Fig 2

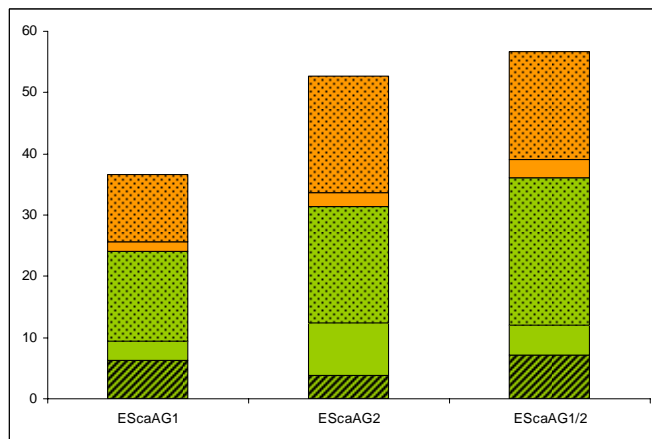

Supplement: Additional file 2 — Supplemental Figure 2: Phenotypes observed in the gynoecium of EScaAG VIGS treated plants. The Y-axis denotes the percentages of different carpel identity phenotypes obtained by VIGS (pTRV2-EScaAG1, n = 239; EScaAG2, n = 209, EScaAG1/2, n = 261 flowers). Differently treated VIGS plants are shown on the X-axis. The green color indicates the occurrence of flat green gynoecia; the orange color symbolizes flat orange gynoecia. Stripes indicate gynoecia enclosing ovules, plane color indicates a gynoecium lacking ovules, and the dotted pattern indicates additional organs enclosed by the gynoecium. [file 2041-9139-1-13-S2.PDF]
