# Supplementary material for: Floral homeotic C function genes repress specific B function genes in the carpel whorl of the basal eudicot California poppy (Eschscholzia californica)
Source: EvoDevo. 2010 Dec 1;1:13. doi: 10.1186/2041-9139-1-13 (PMC3012024; doi:10.1186/2041-9139-1-13)
Supplement: Additional file 3 — Supplemental Table 1: Sequences of primers used in this study. [file 2041-9139-1-13-S3.PDF]

Suppl. Table 1: Sequences of primers used in this study

Primer name	Sequence	purpose
VIGSEcAG1A F	(5´-TA*GGATCC*GTGAAATCCACAATTGAGAGG-3´)	EscaAG1 VIGS vector
EcAG1VIGS R	(5´-TA*CTCGAG*GTCATCCCTTCATATTCATTCC-3´)	EscaAG1 VIGS vector
VIGSEcAG2A F	(5´-*GGATCC*CAACAATAGAAAGGTACAAGAAGAC-3´)	EscaAG2 VIGS vector construction
EcAG2VIGS R	(5´-*CTCGAG*GGAGGGATGTCTGCTCTTG-3´)	EscaAG2 VIGS vector
XbaVIGSEcAG1Bfw	(5´-TA*TCTAGA*GATTAGAGAAAGGCATCAGTAG-3´)	EscaAG1/2 VIGS vector construction
EcAG1VIGSXhorev	(5´-TA*CTCGAG*GTCATCCCTTCATATTCATTCC-3´)	EscaAG1/2 VIGS vector
EcoVIGSEcAG2Afw	(5´-TA*GAATTC*TGCTGAAATTGAGCTCATGC-3´)	EscaAG1/2 VIGS vector
EcAG2VIGSXbarev	(5´-TA*TCTAGAG*GAGGGATGTCTGCTCTTG-3´)	EscaAG1/2 VIGS vector
GAPDH QRT Fw	(5'-GCTTCCTTCAACATCATTCC-3')	Reference gene primer for Q RT PCR
GAPDH QRT Rev	(5'-AGTTGCCTTCTTCTCAAGTC-3')	Reference gene primer for Q RT PCR PCR
ACTIN – 136- F	(5' –AAGAGCTCGAAACTGCCAAG- 3')	Reference gene primer with UPL probe from Roche for Q RT PCR
ACTIN - 136- R	(5' –CATCGGGAAGCTCGTAATTT- 3')	Reference gene primer with UPL probe from Roche for Q RT PCR
EcAG1 QRT Fw1	(5'-AGAAGAGGGAGATTGATTTGC-3')	EScaAG1 primer for Q RT PCR
EcAG1QRT Rev1	(5'-AAGTTCCTAGAGTCATAACCAG-3')	EScaAG1 paralogue specific primer for QRT PCR
EcAG2 QRT Fw	(5'-CGAAACTAGATTAGAGAAAGGC-3')	EScaAG2 primer for QRT PCR
EcAG2 QRT Revspan	(5'- CGCTAGAAATCATGTCGTTGTATTCG - 3')	EScaAG2 paralogue specific primer for QRT PCR
EcDEF1- 132 - F	(5'-GGATGGGAGAGGATTTGGAT-3')	EScaDEF1 primer with UPL probe from Roche
EcDEF1- 132- R	(5'-TTCCAGATTTTGCTCAAGACTTC-3')	EScaDEF1 primer with UPL probe from Roche
EcDEF2RTQfor2	(5'-ATTTGGTGGAGGAGATGATGAG-3')	EScaDEF2 primer for Q RT [illegible]
EcDEF2RTQrev2	(5'-TTTTGAAGATTGGGATGGCTA-3')	EScaDEF2 primer for Q RT [illegible]
EcGLORTQfor2	(5'-TCTAGCACTGGCAAGATGTC-3')	EcGLO primer for Q RT PCR
EcGLORTQ rev2	(5'-TTGATTCTATCCACTTCAGCAC-3')	EcGLO primer for Q RT PCR
